# Dieulafoy Lesion: Scope it Until You Find it

**DOI:** 10.7759/cureus.36097

**Published:** 2023-03-13

**Authors:** Alexander Kusnik, Mostafa Reda Mostafa, Rutwik Pradeep Sharma, Ari Chodos

**Affiliations:** 1 Internal Medicine, Unity Hospital, Rochester Regional Health, Rochester, USA

**Keywords:** computer tomography, egd, hemorrhage, gastrointestinal bleed, dieulafoy

## Abstract

A Dieulafoy lesion is an important consideration every gastroenterologist and internal medicine physician has to make in cases of recurrent, unidentifiable, and hemodynamically compromising gastrointestinal (GI) bleeding. A Dieulafoy lesion is an aberrant vessel that does not reduce in caliber when it extends from the submucosa to the mucosa. Damage to this artery can result in severe, intermittent arterial bleeding from tiny, difficult-to-visualize vessel stumps. Furthermore, these catastrophic bleeding episodes frequently result in hemodynamic instability and the need for transfusion of multiple blood products. As the patients presenting with Dieulafoy lesions often have concomitant cardiac and renal disease, familiarity with this condition is relevant as these patients are at risk of transfusion-related injuries. This case is unique as the Dieulafoy lesion was not visualizable in a standard location despite multiple esophagogastroduodenoscopy (EGD) and CT angiography, illustrating the difficulty of accurately managing and diagnosing this condition.

## Introduction

A Dieulafoy lesion accounts for only 1%-2% of acute all gastrointestinal (GI) bleeds [1], and the mortality rate lies between 9% and 13% [2], with some reports going as high as 80% [3-4]. Despite the advent of endoscopic therapy, the nature of these massive intermittent arterial bleeds commonly results in the obscuration of the active bleeding source, making diagnosis particularly difficult. Furthermore, the lack of surrounding mucosal ulceration and small lesion size contributes to the diagnostic challenge necessitating frequently repeated diagnostic modalities and endoscopic procedures for accurate diagnosis. Among the common pitfalls, endoscopists may also misinterpret coexisting gastritis as the primary source of bleeding. Hence, this topic is also relevant to internal medicine physicians, and providers as they often function as the primary team for patients with intermittent, hemodynamically compromising GI bleeds necessitating the transfusion of multiple blood products.

## Case presentation

**Figure 1:**
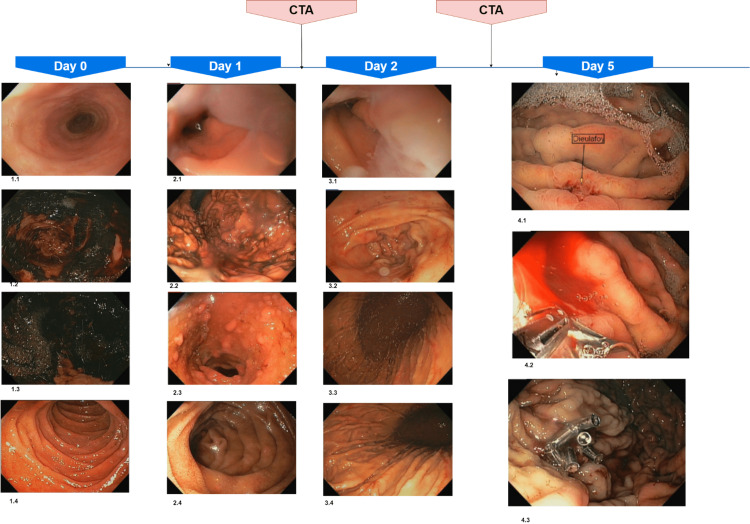
Timeline and number of upper endoscopic procedures required to identify the Dieulafoy lesion. CTA was performed between day 1 and 2, and day 4. 1.1 – esophagus, 1.2 – gastric antrum, 1.3 – gastric fundus, 1.4 –Second part of the duodenum 2.1 – esophagus, 2.2 – gastric fundus, 2.3 – duodenal bulb, 2.4 Second part of – duodenum 3.1 -  GE junction 3.2 – gastric antrum, 3.3 – gastric fundus, 3.4 – gastric body 4.1 – Dieulafoy in mid-gastric body, 4.2 – bleeding Dieulafoy vessel, 4.3 – status post local epinephrine and endoclips CTA, CT angiography

A 65-year-old male with a past medical history significant for heart failure with preserved ejection fraction, non-insulin-dependent diabetes mellitus type II, hypertension, and partial left nephrectomy, presented with upper abdominal pain and melenic stools. The patient embraced the use of non-steroidal anti-inflammatory drugs, including acetylsalicylic acid and ibuprofen, for chronic lower extremity pain. He was found hypotensive but responsive to IV volume resuscitation. The patient received empirically IV pantoprazole and was immediately taken to emergent endoscopy. Endoscopy did not reveal signs of acute bleeding in the duodenum. Despite pre-procedural erythromycin, the gastric examination remained limited due to adherent blood clots and blood pooling in the stomach (Figure 1, images 1.1-1.4). Afterward, the frequency of melenic stools seemed to decrease throughout the night. A mild drop in hemoglobin was initially attributed to IV volume replacement. The patient was admitted to the intensive care unit for closer observation as the blood pressure remained softer and did not improve despite ongoing volume replacement. Due to concern about persistent bleeding, the patient was taken for a repeat upper endoscopy revealing no signs of an active bleed similar to the first endoscopy. Only old adherent blood clots were visualized in the stomach; despite gentle local clot removal, no bleeding was evocable (Figure 1, images 2.1-2.4). Over the following hours, there were no more melenic bowel movements, and close monitoring was continued in the intensive care unit (ICU). The next night, the patient had a recurrence of melenic bowel movements with associated hemodynamic instability; therefore, he was urgently taken for a CT angiogram (CTA). Unfortunately, this examination remained unremarkable, and no active bleed was identified. The patient continued to bleed, requiring multiple transfusions, and was ultimately taken for an emergent third endoscopy. Despite good visualizations of all parts of the upper GI part, no active source of bleeding was identified (Figure 1, images 3.1-3.4). Ongoing bleeding prompted a second CTA, indicating a tiny focus of active hemorrhage in the right lower quadrant during the venous phase, not indicative of the active bleeding source. Surgery was consulted for consideration of potential surgical intervention, but available imaging did not specify what portion of the GI tract was potentially involved; no surgical intervention was possible or indicated. The patient was scheduled for push enteroscopy and capsule endoscopy to further assess a possible small intestinal bleed. The evaluation of the small intestine proved unnecessary as a visible vessel between two gastric rugae was visualized. This finding was consistent with a Dieulafoy lesion located in the gastric body halfway between the folds of the stomach (Figure 1, image 4.1). A brisk arterial bleeding began after placing one endoclip at the site. Five more endoclips were necessary to achieve hemostasis, together with 10 mL of 1:10.000 epinephrine in the surrounding submucosa. The patient required a transfusion of 15 units of blood, four fresh frozen plasma units, and two units of platelet concentrate throughout this hospitalization.

## Discussion

The underlying case underscores a fundamental source of obscure GI bleeding, which is routinely challenging to visualize and is likely underrecognized [5]. A Dielaufoy lesion is also referred to as a “caliber-persistent artery.“ This abnormally large artery fails to decrease in size while it extends into the submucosa and mucosal surface through minor mucosal defects. Approximately half (51%) of all patients with this lesion present with hematemesis and melena together, less than 20% with melena alone [2, 6]. Patients with a Dieulafoy lesion tend to develop acute recurrent arterial bleeding, which commonly requires multiple endoscopic procedures [7-8], as the bleeding vessel is only identified in around 70% of cases during the first endoscopy. Almost one-fifth of the patient requires surgical intervention with explorative laparotomy [2, 4]. As seen in our case, old blood and adherent blood clots decrease the probability of adequately visualizing a Dieulafoy lesion (Figure 1, images 1.2-1.3). Our patient fulfilled the classic patient profile regarding risk factors: males are twice as likely to be affected as females and are significantly affected within the sixth or seventh decade of life [5, 9]. Other commonly observed risk factors include chronic kidney and liver disease [10] and cardiovascular diseases like hypertension [11]. These risk factors and increased age augment the risk of mucosal irritation and localized ischemia, disclosing the irregular artery and associated hemorrhage. Frequent use of nonsteroidal anti-inflammatory drugs (NSAIDs) and anticoagulants is not a risk factor per se. Still, NSAIDs could potentially result in local mucosal injury revealing the aberrant vessel, although more data are needed to confirm this theory [5, 12]. The usually pre-existing risk factors are especially troublesome in the context of ongoing blood loss and the requirement for multiple blood transfusions [7], which enhances the risk of volume overload and other transfusion-associated injuries such as transfusion-associated circulatory overload (TACO) or transfusion-related acute lung injury (TRALI) [13]. Multiple modalities are often necessary to accurately diagnose this condition; Chak et al. [14] indicated in a retrospective study that the diagnostic efficacy of an esophagogastroduodenoscopy (EGD) in Dieulafoy lesions is considerably inferior to that of approximately 95% of other lesions resulting in upper GI bleeding. In our case report, the patient received two CTAs and three EGD without any diagnostic yield. The patient was, therefore, considered for surgical (e.g., wedge resection) and radiological (e.g., angiographic embolization) intervention, but as no precise lesion localization was established, further interventions were deferred. Furthermore, adherent blood clots were accurately visualized in the upper GI tract. Therefore, a diagnostic approach via push enteroscopy was tried, which allows to visualize the full extent of the duodenum and jejunum if needed. A regular EGD is usually sufficient to diagnose Dieulafoy lesions, as almost 75% of these lesions are localized within the stomach, most commonly along the lesser curvature [5]. Other sites include the duodenum (15%), esophagus (8%), and lower GI tract (5%) [2]. Extraintestinal regions can be similarly affected, especially the bronchi, which can present with recurrent hemoptysis in chronic smokers [13]. Other diagnostic modalities, such as capsule endoscopy and CTA, are valuable tools in visualizing Dieulafoy lesions, mainly if the lesion is located within the lower GI system. There are no specific diagnostic criteria for recognizing a Dieulafoy lesion on angiography, and the accurate diagnosis requires an active bleed with associated extravasation from a tortuous vessel. Although no guidelines exist for treating Dieulafoy lesions, an endoscopic procedure is preferred in lesions affecting the upper GI system [5]. As an embolization carries a significant risk for ischemia, it is only recommended if endoscopic therapy fails or a lower GI bleed is located outside the range of therapeutic endoscopy. Finally, as a small, protuberant vessel within the gastric folds was visualized, six endoclips and an epinephrine injection in the surrounding submucosa were necessary to achieve hemostasis (Figure 1, image 4.3). Regarding treatment options, endoscopic methods are considered the treatment of choice compared to surgical and radiological interventions and are most likely responsible for the excellent detection and improved mortality rate, the latter decreasing from 80% to 9%-13% [2] within the last decades which is attributed to the advent of endoscopy. Furthermore, combined endoscopic therapies (e.g., local epinephrine and endoclips) are encouraged as monotherapy burdens an up to 40% higher risk for rebleeding, highlighting the pulsatile character of this lesion [10, 15]. The patient did not experience any other episodes of melena after successful therapy.

## Conclusions

A Dieulafoy lesion is an aberrant vessel that can result in a catastrophic GI bleed. This entity is underrecognized among gastroenterologists and internal medicine physicians, and proper identification is crucial to decrease morbidity. A Dieulafoy lesion represents a critical differential diagnosis in cases of obscure GI bleeding, which frequently necessitates multiple transfusions of blood products, resulting in a high risk of volume overload or other transfusion-associated injuries, especially in patients with already pre-existing cardiovascular diseases. Endoscopic treatment options remain the treatment of choice for Dieulafoy lesions. Still, the obscure and intermittent bleeding character of Dieulafoy lesions often necessitates consideration of other diagnostic modalities, such as CT angiography and capsule endoscopy. The familiarity of this lesion and the proper identification of its drastic and recurring bleeding character might help facilitate correctly diagnosing this condition.
